# Cytokine Expression in Dengue Fever and Dengue Hemorrhagic Fever Patients with Bleeding and Severe Hepatitis

**DOI:** 10.4269/ajtmh.19-0487

**Published:** 2020-03-02

**Authors:** Hisham Ahmed Imad, Weerapong Phumratanaprapin, Benjaluck Phonrat, Kesinee Chotivanich, Prakaykaew Charunwatthana, Sant Muangnoicharoen, Srisin Khusmith, Terapong Tantawichien, Juthamas Phadungsombat, Emi Nakayama, Eiji Konishi, Tatsuo Shioda

**Affiliations:** 1 Department of Clinical Tropical Medicine, Faculty of Tropical Medicine, Mahidol University, Bangkok, Thailand;; 2 Department of Microbiology and Immunology, Faculty of Tropical Medicine, Mahidol University, Bangkok, Thailand;; 3 Division of Infectious Diseases, Department of Medicine, Chulalongkorn University, Bangkok, Thailand;; 4 Mahidol-Osaka Center for Infectious Diseases, Faculty of Tropical Medicine, Mahidol University, Bangkok, Thailand;; 5 Research Institute for Microbial Diseases, Osaka University, Osaka, Japan;; 6 BIKEN Endowed Department of Dengue Vaccine Development, Faculty of Tropical Medicine, Mahidol University, Bangkok, Thailand

## Abstract

Dengue is the most common mosquito-borne flaviviral infection in the world today. Several factors contribute and act synergistically to cause severe infection. One of these is dysregulated host immunological mediators that cause transient pathophysiology during infection. These mediators act on the endothelium to increase vascular permeability, which leads to plasma leakage compromising hemodynamics and coagulopathy. We conducted a prospective study to explore the expression of pro- and anti-inflammatory cytokines and how they relate to clinical dengue manifestations, by assessing their dynamics through acute dengue infection in adults admitted to the Hospital for Tropical Diseases, Bangkok, Thailand. We performed cytokine analysis at three phases of infection for 96 hospitalized adults together with serotyping of confirmed dengue infection during the outbreaks of 2015 and 2016. The serum concentrations of seven cytokines (interleukin [IL]-2, IL-4, IL-6, IL-8, IL-10, tumor necrosis factor alpha, and interferon gamma) were measured in duplicate using a commercial kit (Bio-Plex Human Cytokine Assay). In this study, the cytokine profile was suggestive of a T-helper 2 response. Most patients had secondary infection, and the levels of viremia were higher in patients with plasma leakage than those without plasma leakage. In addition, we observed that bleeding and hepatitis were associated with significantly higher levels of IL-8 during the early phases of infection. Furthermore, IL-6 levels in the early phase of infection were also elevated in bleeding patients with plasma leakage. These results suggest that IL-6 and IL-8 may act in synergy to cause bleeding in patients with plasma leakage.

## INTRODUCTION

The dengue virus is a *flavivirus* of the family Flaviviridae and has been characterized into four serotypes (Dengue viruses 1 to 4). Transmitted by *Aedes* species, dengue infection is the most commonly occurring arthropod-borne viral infection globally. Several factors such as globalization, urbanization, and lack of effective vector control have facilitated the spread of this disease beyond the subtropics.^1^

It is estimated that 50–400 million infections occur annually. In Thailand, the first epidemic of dengue hemorrhagic fever (DHF), a severe form of dengue virus infection, occurred in 1958. Multiple outbreaks with greater epidemic potential have occurred since then, with a range of 15,000–105,000 cases occurring each year in Thailand.^2^ The infection causes a systemic viral infection, and complications are associated with the pathogenic role of endothelial cells.^3–5^ Cytokines^6,7^ increase vascular permeability and hemorrhage during dengue infection. These molecules are proteinaceous and are secreted during innate and adaptive immunological responses, acting as inflammatory mediators or modulatory molecules during dengue infection. Viral infection activates the innate immune response to express pro-inflammatory cytokines that recruit and activate cells involved in inflammation and the induction of adaptive immunity. Cytokines also help distinguish between T-helper 1 and T-helper 2 (Th2) cells, and their expression in dengue has been reviewed extensively.^8^ The dengue virus is tropic and replicates in human monocytes, macrophages, and hepatocytes, which are known to express cytokines.^9^

The endothelium plays a pivotal role in the pathophysiology of dengue infection.^6^ Activation of the endothelium by the expression of interleukin (IL)-6, IL-8, and tumor necrosis factor alpha (TNFα) have been previously described.^3,10^ This activation of the endothelial system not only contributes to the bleeding, which is common in dengue fever (DF) and DHF,^11^ but also to liver damage, leading to hepatitis complicating dengue.^12^ Bleeding manifestations are seen in 20–60%^13^ of cases, and hepatitis with a 100-fold increase in transaminase was reported in 7% of cases.^14^ Thrombocytopenia with impairment of the coagulation system is associated with bleeding in dengue,^15^ and multiple factors contribute to the development of hepatitis. These factors include hypoxic injury caused by decreased perfusion, direct damage by the virus, and dysregulated immune-mediated injury in response to dengue virus. Interleukin-8 is expressed by hepatocytes during dengue infection,^16^ with reports of cytokines correlating with elevated transaminase levels^17^ and the expression of TNFα following the apoptosis of hepatocytes in dengue infection.^18^ Others reported a correlation between severity and IL-6 and TNFα levels.^19^ However, only limited data are available for the relationship between the increased expressions of cytokines with hepatitis and bleeding. In the present study, we investigated the expressions of cytokines during dengue infection with bleeding and hepatitis and assessed the degree of viremia with plasma leakage.

## MATERIALS AND METHODS

This was a prospective study of hospitalized adults with symptomatic dengue infection at the Hospital for Tropical Diseases, Bangkok, Thailand. Ethical approval to conduct this study was obtained from the Ethics Committee, Faculty of Tropical Medicine, Mahidol University.

During the dengue outbreak in 2015 and 2016, we recruited adults who presented within 5 days after developing symptoms and with a positive non structural protein 1 antigen or a positive anti-dengue IgM test into our study. We excluded any vulnerable groups and participants who had received a transfusion of blood products during the study period. Because this was an observational study, all consenting participants meeting the study criteria were recruited during the 2-year study period. The participants were grouped based on the WHO 1997 classification, and a chest X-ray was performed to detect pleural effusion. Complications in dengue infection included bleeding and severe hepatitis (aspartate aminotransferase/alanine aminotransferase > 400 U/L).

### Cytokine measurement.

Blood specimens were collected for cytokine analysis during the three phases of infection. A commercial assay (Bio-Plex Human Cytokine Assay; Bio-Rad Inc., Hercules CA) was performed to detect the levels of IL-2, IL-4, IL-6, IL-8, IL-10, interferon gamma (IFNγ), and TNFα. In brief, sera obtained from participants during the three phases were mixed with beads coated with antibodies to cytokines and a unique fluorescent intensity. Subsequently, the mixtures were incubated with biotinylated anti-cytokine antibodies. Finally, phycoerythrin-conjugated streptavidin was added, and the fluorescent signals were detected using a multiplex array reader (Bio-Plex 200 System, Bio-Rad Inc., Hercules, CA). Raw data were initially measured as the relative fluorescence intensity and then converted to cytokine concentration based on a standard curve generated from the reference concentrations.

The acute phase specimen corresponded to blood collected within 5 days following the development of symptoms of dengue infection. The defervescence specimens were obtained 8 hours after the participants had remained afebrile with a recorded body temperature of 37.7°C or lower. The convalescence specimens were obtained during the follow-up within or later than 2 weeks after the onset of symptoms. The standard provided by the manufacturer was used as a control, and the observed readings were determined as the detection limits in pg/mL.

### Quantification of RNA and sequence of infection.

To determine the dengue serotype, a commercially available dengue subtyping multiplex kit by the Genesig company (Chandler’s Ford, United Kingdom) was used as per the manufacturer’s instruction.^20^ Then, RNA quantification was performed using a One-Step SYBR^®^ Prime Script RT-PCR Kit II, Takara Bio Inc., (Kusatsu, Japan); cDNA synthesis from RNA was performed using reverse transcriptase Prime Script RTase; and polymerase chain reaction amplification was performed by TaKaRa Ex Taq HS. This technique was previously described.^21^ For dengue serology, commercially available dengue IgM and IgG immunochromatography kits from Panbio Dengue Duo Cassette Abbott Inc., (Chicago, IL) were used in accordance with the manufacturer’s protocol. A serum aliquot representing the day of defervescence was used in these test kits. Primary infections were diagnosed when only anti-dengue IgM was positive together with the appearance of the control band. Secondary infections were determined when both anti-dengue IgM and IgG were positive, including the appearance of the control band.

### Statistical analysis.

The Mann–Whitney *U*-test was used to assess differences in the cytokine levels between DF and DHF. To establish the correlation between cytokine levels and clinical parameters/findings, a correlation matrix was applied. Results are given as the correlation coefficient; *r* (range, from −1 to +1). A two-tailed *P*-value < 0.05 was considered significant for all tests performed. One-way analysis of variance was used to evaluate differences among cytokine levels in the different groups. All statistical analyses were performed using Microsoft Excel, SPSS version 18, and GraphPad Prism 7 for Windows, version 7.30 (San Diego, CA).

## RESULTS

In this study, 96 hospitalized adult patients with a confirmed dengue infection were recruited during the outbreak in 2015 and 2016. The demographic and clinical data as well as laboratory parameters of the study population and clinical details are shown in Table 1. Our results indicate that symptoms of systemic illness including headache (*P* = 0.02), myalgia (*P* = 0.03), lymphadenopathy (*P* = 0.02), and bleeding (*P* = 0.05) were more common in DHF. A longer duration of hospitalization was observed in DHF cases than DF cases (*P* = 0.01). The hematological profile was typical for dengue infection, with leukopenia and thrombocytopenia (platelet count < 100 × 10^3^/µL) being obvious findings in our subjects. From the analysis of liver function tests, transaminitis was observed in 11% of subjects. From the analysis of liver function tests, transaminitis was observed in 11% of subjects. The mean ± SD levels of aspartate aminotransferase and alanine aminotransferase for DF were 224 ± 399 U/L and 127 ± 192 U/L, and for DHF were 308 ± 528 U/L and 122 ± 184 U/L, respectively.

**Table 1:** Demographic data, clinical findings, and laboratory parameters in dengue infection

Parameters	Dengue fever = 59, *n* (%)	Dengue hemorrhagic fever = 37, *n* (%)	*P*-value
Females	20 (33.89)	18 (48.64)	0.2
Males	39 (66.10)	19 (51.35)	
Age (years), mean ± SD	31 ± 11	33 ± 11	0.44
Primary infection, age mean ± SD	25 ± 11	0	NA
Secondary infection, age mean ± SD	32 ± 11	33 ± 11	0.11
Duration of illness, mean ± SD	6.95 ± 0.9	7.8 ± 0.8	**0.01**
Fever	59 (100)	37 (100)	NA
Headache	53 (89.83)	37 (100)	**0.02**
Myalgia/arthralgia	43 (72.88)	34 (91.89)	**0.03**
Nausea/vomiting	29 (49.15)	20 (54.05)	0.75
Lymphadenopathy	21 (35.59)	22 (59.45)	**0.02**
Hepatomegaly	18 (30.50)	15 (40.50)	0.31
Bleeding	20 (32.78)	20 (54.05)	**0.05**

	Mean ± SD	Mean ± SD	*P*-value
Hemoglobin (g/dL)	14.3 ± 1.3	14.2 ± 1.9	0.53
Hematocrit (%)	42.4 ± 3.8	41.9 ± 5.3	0.54
Leukocytes (10^3^/µL)	3.6 ± 1.7	3.4 ± 1.5	0.5
Neutrophils (%)	50.7 ± 16.5	51.3 ± 16.8	0.85
Lymphocytes (%)	26.7 ± 12.5	25.3 ± 11.6	0.58
Platelets (10^3^/µL)	81 ± 46	63 ± 43	0.06
Atypical lymphocytes (%)	14 ± 10	13 ± 12	0.75
Sodium (mmol/L)	136 ± 3	134 ± 2	0.06
Potassium (mmol/L)	4.2 ± 3.9	3.7 ± 0.4	0.85
Bicarbonate (mmol/L)	23.7 ± 2.3	23.9 ± 2.9	0.71
Blood urea nitrogen (mg/dL)	10.1 ± 5.0	11.2 ± 4.6	0.23
Creatinine (mg/dL)	0.9 ± 0.3	0.9 ± 0.3	0.9
Total protein (g/dL)	6.9 ± 0.5	8.9 ± 12.7	0.25
Albumin (g/dL)	3.9 ± 0.3	3.9 ± 0.5	0.53
Total bilirubin (mg/dL)	0.5 ± 0.2	0.6 ± 0.5	0.14
Direct bilirubin (mg/dL)	0.19 ± 0.1	0.3 ± 0.3	0.16
Aspartate transaminase (U/L)	224 ± 399	308 ± 528	0.24
Alanine transaminase (U/L)	127 ± 192	122 ± 184	0.93

NA = not applicable. The data in the table represent the demographic data and clinical findings in DF and DHF. The clinical findings are represented as the actual number and percentage. The laboratory parameters are represented as the median ± SD. The chi-square test was used to determine the differences in clinical findings in both groups. The Mann–Whitney U-test was used to determine the *P*-value between two groups using the median value. Bold indicates statistically significant *P*-values.

### Cytokine expressions during dengue infection.

**Figure 1.**
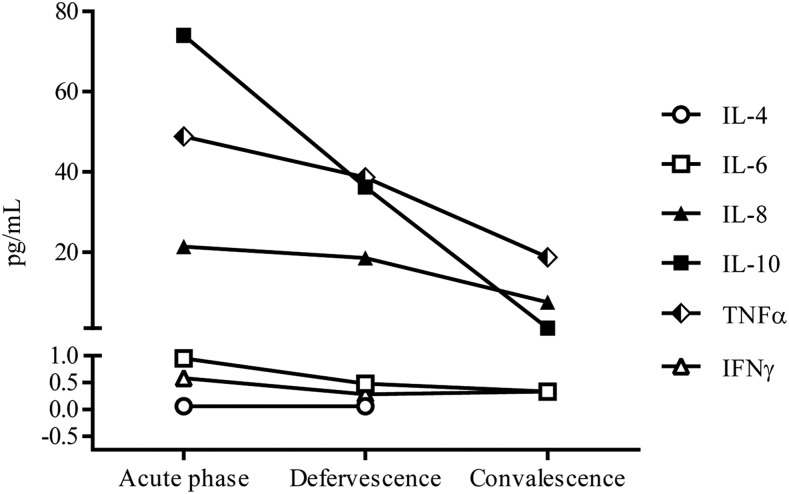
Cytokine expression during the three phases of dengue infection.

**Figure 2.**
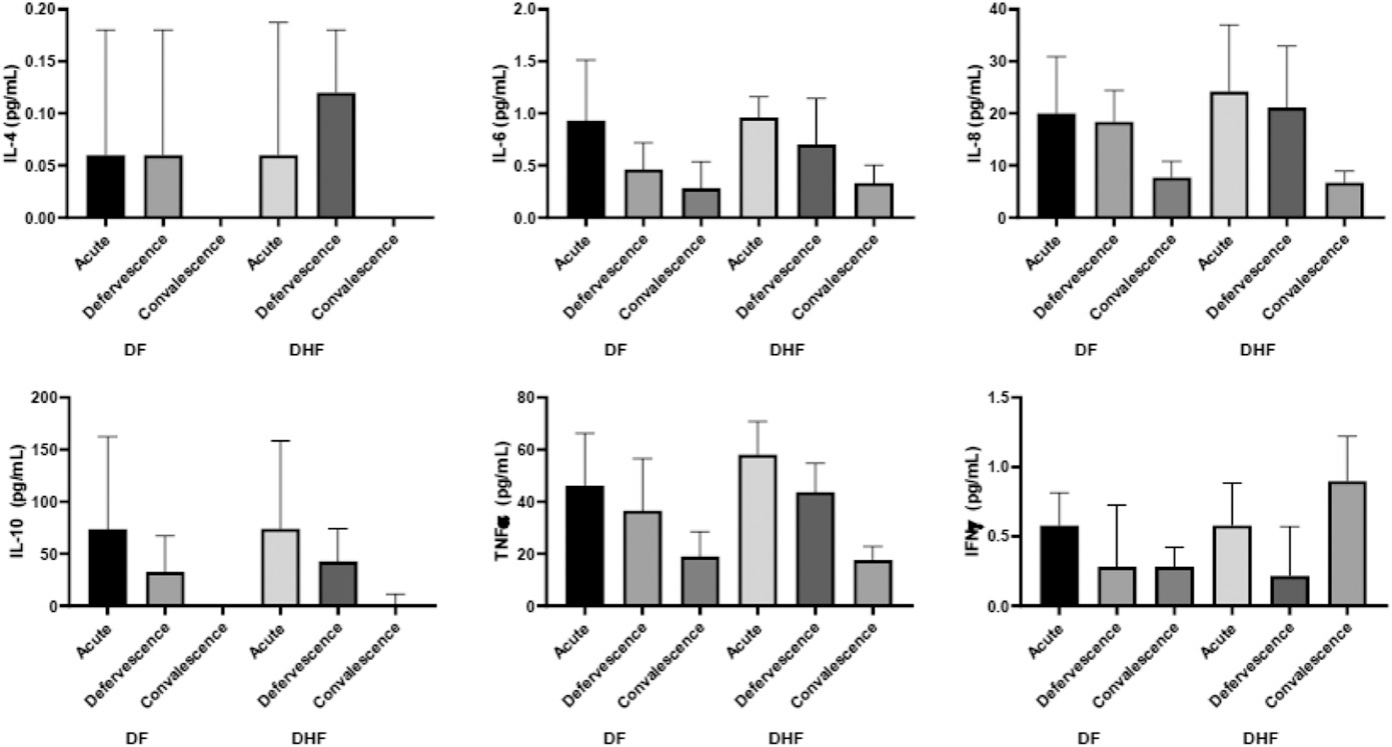
Cytokine profile during the three phases of infection dengue fever vs. dengue hemorrhagic fever.

**Figure 3.**
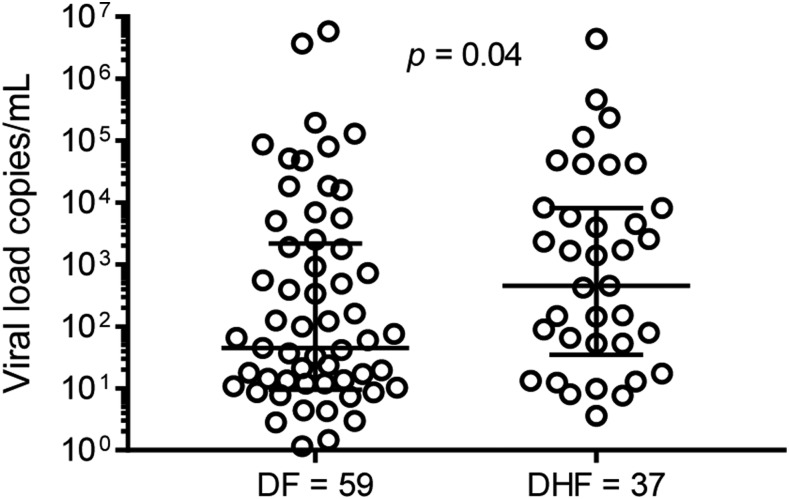
Viral load during the acute phase in dengue fever and dengue hemorrhagic fever.

Figure 1 shows the trend of the expressions of IL-4, IL-6, IL-8, IL-10, TNFα, and IFNγ during the three phases of dengue infection. The expressed cytokines peaked during the acute phase. Interleukin-10 was most expressed during the acute phase followed by TNFα, IL-8, IL-6, IFNγ, and IL-4. Interleukin-2 was not detectable. At defervescence, TNFα was the most expressed, and during convalescence, IL-4 had diminished to low levels and was undetectable in most cases. We investigated the cytokine profile by grouping our subjects based on their clinical classification. These included 59 subjects with DF and 37 with DHF, of whom 17 were of DHF grade I, 19 were of DHF grade II, and a single participant was of DHF grade IV as per the WHO 1997 classification.^22^ Most patients had secondary dengue infection and had an uneventful milder clinical course during hospitalization. Complications identified in our study were shock in a single subject, and bleeding in 42% and hepatitis in 11% of study subjects. The bleeding manifestations were mucosal bleeding in 31.6%, menorrhagia in 27.5%, epistaxis in 5.1%, melena in two subjects, and hematemesis in a single subject. Our results revealed no significant differences in cytokine levels during the three phases of infection between subjects with and without plasma leakage (Figure 2). Nevertheless, we did observe higher viremia in those with plasma leakage (Figure 3).

### Cytokines profile with bleeding.

**Figure 4.**
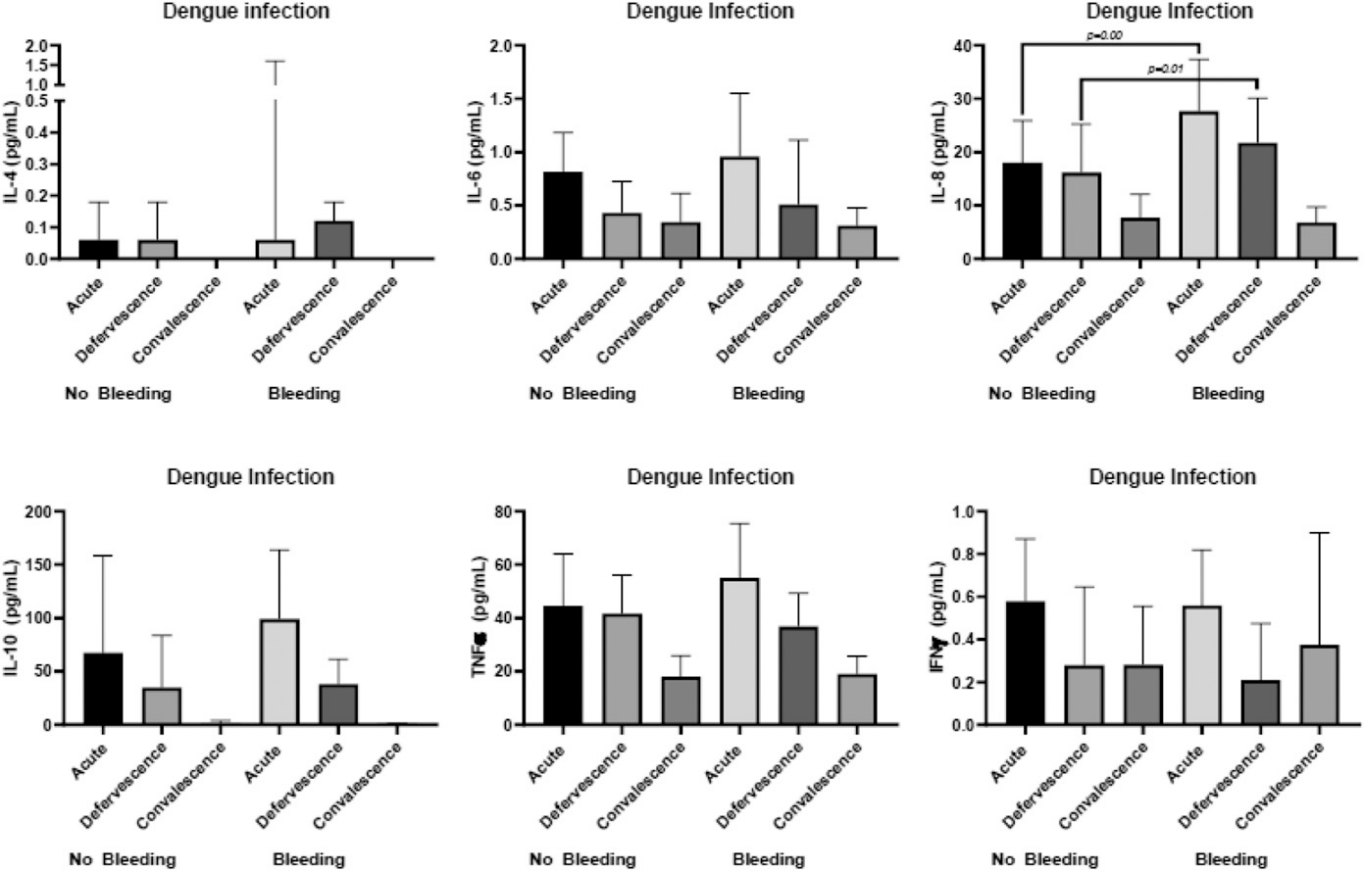
Cytokine profile for bleeding during the three phases of infection.

Interleukin-8 expression was significantly elevated during the acute phase and at defervescence. The IL-8 median value (interquartile range) was 27.65 (range, 17.42–37.42) pg/mL for the group with bleeding during the acute phase and 17.98 (range, 13.06–25.95) pg/mL for the group without bleeding (*P* = 0.00, Figure 4). Similarly, at defervescence, the IL-8 median value (IQR) was 21.76 (range, 16.86–30.14) pg/mL for the group with bleeding and 16.19 (range, 11.18–25.30) pg/mL for the group without bleeding (*P* = 0.01, Figure 4). These data represent the detectable levels of cytokines in 96 subjects during the three phases of dengue infection. The cytokine levels are shown as the median and IQR in pg/mL. The Mann–Whitney U-test was used to determine the *P* value between two groups using the median value.

**Figure 5.**
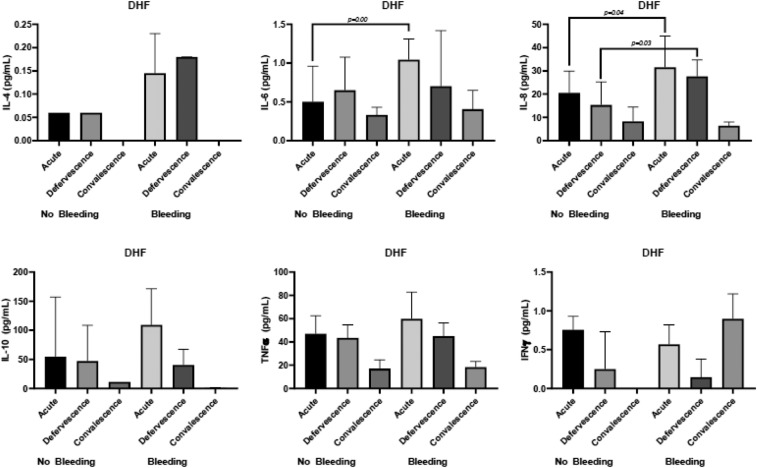
Cytokine profile for bleeding in dengue hemorrhagic fever during the three phases of infection.

**Figure 6.**
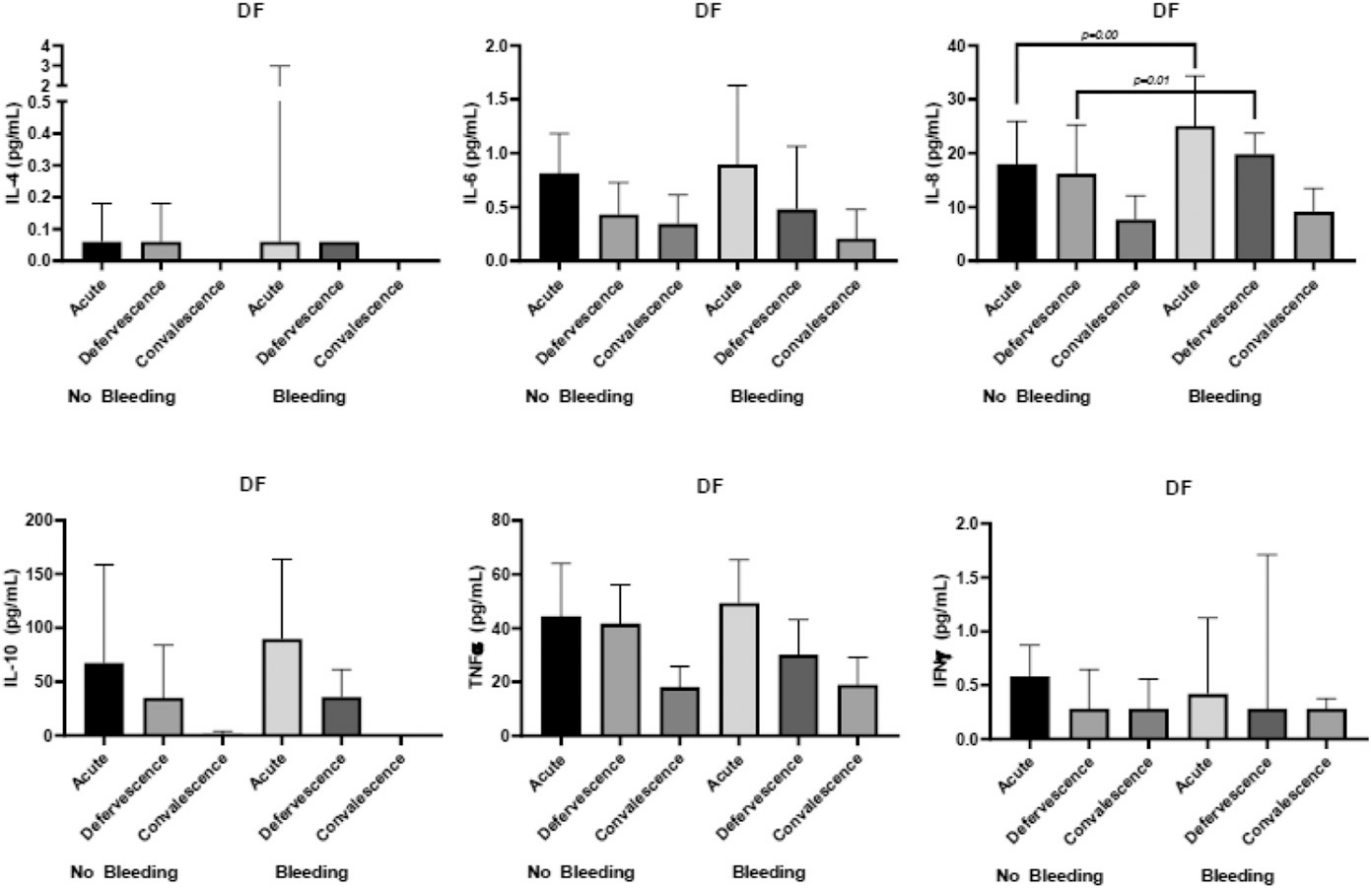
Cytokine profile for bleeding in dengue fever during the three phases of infection.

Similarly, the levels of IL-8 were significantly higher in DHF grade II–IV patients than DHF grade I patients (Figure 5). In addition, IL-6 levels during the acute phase were significantly elevated in DHF grade II–IV compared with DHF grade I (Figure 5). The levels of IL-8 were also significantly higher in DF with bleeding than DF without bleeding during the acute phase of infection (Figure 6).

### Cytokine profile with hepatitis.

**Figure 7.**
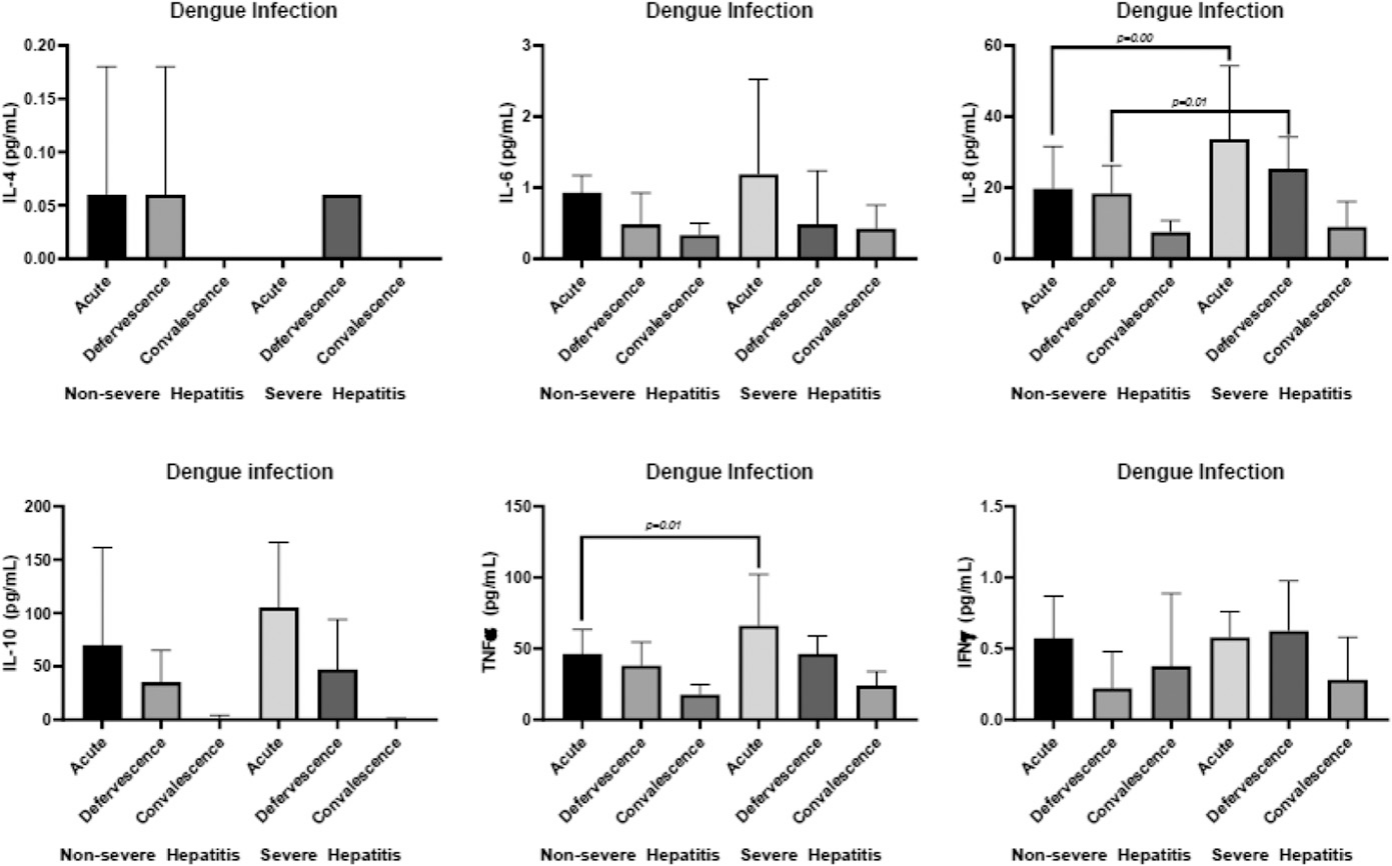
Cytokine profile during the three phases of infection for severe hepatitis (AST/ALT ≥ 400 U/L).

In the cytokine profile generated for severe hepatitis (AST/AST > 400 U/L), we observed that in addition to IL-8 being significantly elevated during the acute phase (*P* = 0.00) and at defervescence (*P* = 0.01), TNFα was significantly elevated during the acute phase (*P* = 0.01, Figure 7).

The levels of IL-8 were significantly higher during the acute phase and at defervescence in patients with an increase in liver enzymes (AST/ALT ≥ 1,000 U/L). The median value of IL-8 was 30.28 pg/mL (IQR, 25.18–86.58) in subjects with AST/ALT ≥ 1,000 U/L, which was significantly elevated during the acute phase (*P* = 0.03). At defervescence, in subjects with AST/ALT ≥ 1,000 U/L, the median IL-8 value was 32.29 pg/mL (IQR, 23.80–57.67), which was significantly elevated compared with subjects with AST/ALT < 1,000 U/L (*P* = 0.01).

## DISCUSSION

In the present study, the levels of cytokines during dengue infection were measured, and we analyzed the profiled cytokine patterns to determine the immuno-pathophysiological events that occur during dengue infection leading to complications, such as bleeding and hepatitis. To the best of our knowledge, few studies have performed cytokine analysis during the three phases of infection. In particular, we designed our study to measure the cytokine levels at defervescence and identified elevated cytokine levels with complications. In viral infections, cytokines can interfere with the interferon signaling pathway,^23^ which is the host’s primary antiviral response to an acute infection.^24^ Our study subjects had a diminished IFNγ response, which might have resulted from a secondary dengue infection, where the Th1 response is prominent in primary infection.^25^ This phenomenon was previously demonstrated in vitro, where dengue virus–infected cells failed to express or demonstrated a reduced expression of IFNγ on secondary infection.^26^ The cytokine response reflecting a T-helper 2 response was supported by our undetectable levels of secreted IL-2, in addition to the very low levels of IFNγ, with an increased expression of IL-10. This trend of cytokine expression also suggests immunoregulatory effects consistent with secondary infection.^27^ Memory B cells are primed to produce antibodies by the expression of IL-10,^28^ and with its proteolytic properties, IL-10 inhibited IFNγ expression and deregulated the expressions of IL-6, IL-8, and TNFα in our study subjects and as previously described.^29^ Unlike previous reports that showed an association of IL-4 and disease severity,^30^ our study failed to reveal such findings probably because IL-4 was detected in only a few cases partly because of the small number of study subjects.

We demonstrated endothelial activation by the detection of IL-6, IL-8, and TNFα as previously described.^3,10^ This activation of the endothelial system not only contributes to bleeding, which is common in DF and DHF,^11^ but also involved in liver damage, leading to hepatitis complicating dengue.^12^ Our results were consistent with previous studies in respect to higher viremia in DHF,^31^ and we observed more bleeding and symptoms of systemic illness in DHF, reflecting the degree of severity when compared with DF. However, we did not find any association between viremia and bleeding as previously reported.^32^ Interleukin 8 is expressed by hepatocytes in dengue infection^16^ and was associated with disease severity.^17^ Similar to a previous study,^33^ our results regarding IL-8 levels could not distinguish DF from DHF. However, we demonstrated significantly elevated levels of IL-8 during the acute phase and at defervescence in patients with bleeding and in those with hepatitis. We also showed an association of an increased IL-8 level with bleeding during both phases. Significantly, elevated levels of IL-6 were observed during the acute phase in patients with bleeding, with a background of plasma leakage, whereas others reported elevated levels of IL-6 in DHF^34^ and bleeding.^32^ Other studies reported TNFα was not detectable or only detected in 30% of cases of dengue infection.^35^ In our study, we detected TNFα expression in all our subjects. We highlighted other factors that contribute to bleeding in dengue, such as thrombocytopenia,^36^ which can worsen bleeding in dengue infection.

The liver impairment, which occurs in dengue infection,^14^ impacts bleeding with a worsening of coagulation. Our results demonstrated that IL-8 and TNFα expressions were significantly higher in patients with transaminases elevated 10-fold. Furthermore, we observed that IL-8 levels were significantly elevated with a 100-fold increase in transaminase levels during both phases.

The properties of IL-8 contribute to platelet activation and endothelial permeability to cause thrombocytopenia,^34^ which worsens bleeding in dengue infection. We also observed that the platelet counts during both phases were significantly decreased in patients with bleeding. These elevated levels of IL-8 observed in our study might facilitate the events preceding bleeding during dengue infection. A similar observation of elevated levels of IL-8 with thrombocytopenia in dengue was described.^34^ During dengue infection, infected hepatocytes express IL-8, attracting neutrophils to the liver, causing liver injury and impaired coagulation. Our results show increased levels of neutrophils during the two phases of infection with hepatitis. This increase in neutrophils with hepatitis might be a result of the elevated levels of IL-8 with hepatitis. Although a positive correlation of IFNγ and transaminase was reported,^34^ we observed no similar pattern of correlations. Nevertheless, there were positive correlations between IL-6 and IL-8 and between IL-6 and IFNγ during the acute phase, and at defervescence IL-8 and TNFα were positively correlated, indicating these cytokines may act in synergy. In summary, the immunoregulatory responses observed may be implicated in the bleeding and hepatitis observed in dengue patients.

## CONCLUSION

In this study, higher viremia in DHF with a T-helper 2 response determined by profiling the cytokine expression was demonstrated. The expression of IL-8 was significantly elevated during the acute phase in patients with bleeding in both DF and DHF, with a further increase in IL-8 levels in DHF at defervescence. IL-6 acts in synergy with IL-8 for bleeding with plasma leakage during the acute phase. Significantly, higher levels of TNFα, IL-6, and IL-8 were observed in cases of severe hepatitis.

### Limitations.

There were some limitations in this study including the small number of total study subjects and those with severe disease.
